# Reorganization of the Y Chromosomes Enhances Divergence in Israeli Mole Rats *Nannospalax ehrenbergi* (Spalacidae, Rodentia): Comparative Analysis of Meiotic and Mitotic Chromosomes

**DOI:** 10.3390/genes9060272

**Published:** 2018-05-24

**Authors:** Sergey Matveevsky, Elena Ivanitskaya, Victor Spangenberg, Irina Bakloushinskaya, Oxana Kolomiets

**Affiliations:** 1 Vavilov Institute of General Genetics, Russian Academy of Sciences, Moscow 119991, Russia; v.spangenberg@gmail.com (V.S.); olkolomiets@mail.ru (O.K.); 2 Institute of Evolution, Haifa University, Haifa 31905, Israel; lenaiva@research.haifa.ac.il; 3 Koltzov Institute of Developmental Biology, Russian Academy of Sciences, Moscow 119334, Russia; irina.bakl@gmail.com

**Keywords:** *Nannospalax ehrenbergi*, chromosome forms, meiosis, synaptonemal complex, Y chromosome, sex bivalent, asymmetric synapsis, recombination, chromosome evolution

## Abstract

The Y chromosome in mammals is variable, even in closely related species. Middle East blind mole rats *Nannospalax ehrenbergi* demonstrate autosomal variability, which probably leads to speciation. Here, we compare the mitotic and meiotic chromosomes of mole rats. For the first time, we studied the behavior of their sex chromosomes in the meiotic prophase I using electron microscopy and immunocytochemical analysis. Unexpectedly, the sex chromosomes of the 52- and 60-chromosome forms of mole rats showed different synaptic and recombination patterns due to distinct locations of the centromeres on the Y chromosomes. The absence of recombination in the 60-chromosome form, the asymmetric synapsis, and the short-term disturbance in the synaptic co-orientation of the telomeric regions of the X and Y chromosomes were revealed as specific features of mole rat sex bivalents. We suggest several scenarios of Y chromosome alteration in connection with species differentiation in mole rats.

## 1. Introduction

Sex chromosomes are highly specialized elements of the genome that differ from autosomes in terms of specific gene contents, distinct behavior in meiosis, and a variety of evolutionary paths [1]. Sex chromosomes demonstrate an amazing diversity in animals [2], particularly in mammals [3,4,5]. In placental mammals, sex chromosomes evolved over 180 million years and appear to be heteromorphic, X and Y chromosomes, except in some cases of multiple sex chromosomes (several Xs or several Ys, or both) or, extraordinarily, lack of a Y [3,6,7,8]. In comparison with the X chromosome, which is large and rich in genes, the Y chromosome in most mammals is small, heterochromatic, and contains significantly fewer genes [9,10]. The Y chromosome is often subjected to various structural changes and additions.

A genetic linkage for male-specific genes was selected as soon as male-determining genes emerged on a proto-Y chromosome [11,12,13,14,15]. The dark side of the specialization appeared to be a deficiency in recombination due to the absence of homological regions between X and Y chromosomes, the declining force of natural selection, accumulating deleterious mutations, and further degradation of the Y chromosome [16,17,18]. The Y chromosome may disappear, as in four species of mole voles *Ellobius* [4,19,20,21] and some species of spiny mice *Tokudaia* [22]. Several possible ways of slowing down the decay of the Y chromosome were hypothesized, for example by gene duplications, adding repeats, doubling and translocating parts of X, or fusing with autosomes or their parts [23,24,25,26]. If an autosome or some part of it is translocated onto Y, so called ‘neo’ sex chromosomes form, as in spiny mice *Tokudaia muenninki* [27], Indian mongoose *Herpestes auropunctatus* [28,29], and South-American primates *Aotus* [30,31] and *Alouatta* [32,33]. A comparison of Y in different primate species made it possible to conclude that there are pericentric inversions in the early stages of the primates’ evolution [34]. In most mammals, the pseudoautosomal region (PAR) is a short region of homology, allowing pairing and recombination that is required for faithful segregation of the male sex chromosomes [35]. The non-combining part of the human Y chromosome underwent four overlapping inversions, which resulted in a reduction in the PAR length [36]. Inverse polymorphism of the Y chromosome in different human populations [37,38], pericentric inversion in Y in infertile men [39], and neodicentricity of the human Y [40] have been identified.

Middle East, or Palestine, blind mole rats *Nannospalax ehrenbergi* (Nehring, 1898) are especially interesting, because they demonstrate high autosome variability [41,42]. These animals are of particular interest to geneticists, physiologists, and medical researchers. They are distinguished by a unique anti-carcinogenic system, unusual longevity for rodents, adaptation to conditions of hypoxia, and vision reduction [43,44,45,46].

**Figure 1:**
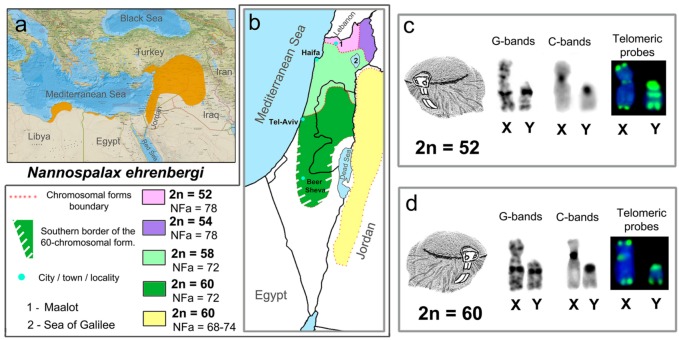
Geographical distribution and sex chromosomes of two chromosomal forms of *Nannospalax ehrenbergi* in Israel. (**a**) The geographic range of the *N. ehrenbergi* superspecies (from the IUCN Red List of Threatened Species; http://www.iucnredlist.org); (**b**) distribution of chromosomal forms of mole rats in Israel and Jordan on the basis of Nevo et al. [48], Qumsiyeh [49], and [51]; (**c**,**d**) sex chromosomes of mole rat forms with 2n = 52, NFa = 78 (**c**) and 2n = 60, NFa = 72 (**d**). Fluorescent in situ hybridization (FISH): telomeric probes (green), DAPI (blue). NFa—number of the autosomal arms.

Populations of *N. ehrenbergi* occupy open areas in southeastern Anatolia, northern Iraq, Syria, Lebanon, Jordan, and Israel, with local coastal populations in Northern Egypt and Libya (Figure 1a). To date, 19 chromosomal forms have been identified in blind mole rats (for a review, see [47]), four of which inhabit Israel: 2n = 52, 2n = 54, 2n = 58, and 2n = 60 [42,48,49] (Figure 1b). In *N. ehrenbergi*, the X chromosome is a mid-length submetacentric with a small block of pericentromeric heterochromatin. The Y chromosome is a small acrocentric, carrying a large pericentromeric block of heterochromatin [48]. Such characteristics of the sex chromosomes are similar to those in other species of the genus *Nannospalax*, although there are some differences between them [50,51]. Mole rats are a fruitful model for studying the co-evolution of sex chromosomes and highly variable autosomes and for estimating the evolutionary consequences of chromosome alterations.

The structure and behavior of the sex chromosomes in meiosis in blind mole rats appears to be almost unknown. Some details of sex chromosome synapsis in a 55-chromosome hybrid male *N. ehrenbergi* were described by Wahrman et al. [42]. There are no other data on the features of synapsis and recombination of the sex chromosomes in blind mole rats. The aim of our work was to conduct a detailed electron-microscopic and immunocytochemical analysis of synapsis and recombination of the sex chromosomes in two forms of *N. ehrenbergi* from Israel and to clarify if alterations in the sex chromosomes could facilitate a divergence of the forms.

## 2. Materials and Methods

### 2.1. Animals

Electron-microscopic and immunocytochemical analyses were carried out in two male blind mole rats, which were caught in the geographic range of the chromosomal form with 2n = 60, NF = 72 (vicinity of Beer Sheva: 31°14′30.8″ N × 34°42′11″ E), and two males with 2n = 52, NFa = 78 which were caught near Miilya village, Upper Galilee (33°01′32.6″ N, 35°14′51.8″ E).

Analysis of the G- and C-banding patterns and of the distribution of telomeric sequences in mitotic chromosomes were also performed for two males of 2n = 60 from Lahav (vicinity of Beer Sheva: 31°22′41.87″ N, 34°52′13.43″ E), two males of 2n = 58 from Muchraka, Mount Carmel (32°44′34.7″ N, 35°02′54.2″ E), one male of 2n = 54 from Al Qunaitra (33°07′15.5″ N, 35°51′04.2″ E), and two males of 2n = 52 from Kerem Ben Zimra (33°01′56.9″ N, 35°28′16.5″ E).

The manipulations of the animals were carried out in accordance with the international rules of the Manual on Humane Use of Animals in Biomedical Research [52] and the rules of the Ethics Committee for Animal Research of the Vavilov Institute of General Genetics (protocol No. 3 from 10 November 2016).

### 2.2. Preparation of Mitotic Chromosomes and Fluorescent In-Situ Hybridization

Mitotic metaphases were obtained from bone marrow cells [53]. Chromosome slides were prepared according to the standard air-drying technique. The C-banding technique was performed as described by Sumner [54]. To obtain G-banded chromosomes, we used a simple two-step method (modified method from Seabright [55]). For the localization of telomeric sequences, we used commercially available telomeric (TTAGGG)n probes labeled with biotin-16-dUTP (Roche Biomolecular, Indianapolis, IN, USA), detected with avidin-FITC (Vector Laboratories, Burlingame, CA, USA). The hybridization was carried out at 37 °C overnight. The chromosomes were counterstained with DAPI.

### 2.3. Preparation of Meiotic Chromosomes

The spreading of meiotic chromosomes was performed according to Navarro et al. [56]. The spermatocytes were spread on a hypotonic solution of sucrose (0.2 M) and fixed in 4% paraformaldehyde (pH 8.2), then washed in 0.4% Photo-Flo solution (Kodak, Rochester, NY, USA), dried, and kept at −20 °C. The spreads were prepared on slides coated with a Falcon film, contrasted with a 50% solution of silver nitrate, and examined under the electron microscope Jem-1011 (Jeol, Tokyo, Japan). For immunocytological analyses, the spreads were prepared on slides with poly-L-lysine.

### 2.4. Antibodies and Immunostaining

All antibodies were diluted in phosphate-buffered saline (PBS) containing 3% bovine serum albumin (BSA) and 0.05% Triton-X-100. We used the following primary antibodies (Abcam, Cambridge, UK): for the immunodetection of axial/lateral elements, rabbit anti-SYCP3 antibody at a 1:500 dilution; for the identification of the central element, rabbit anti-SYCP1 antibody at a 1:500 dilution; for the detection of late recombination nodules, mouse anti-MLH1 antibody at a 1:50 dilution. To identify the kinetochore proteins, we used human CREST antibody at a 1:600 dilution (Fitzgerald Industries International Inc., Concord, MA, USA).

We used the following secondary antibodies: goat anti-rabbit IgG, Alexa Fluor 488-conjugate (Invitrogen, Carlsbad, CA, USA); goat anti-human IgG, Alexa Fluor 555-conjugate (Invitrogen); goat anti-mouse IgG, fluorescein isothiocyanate (FITC)-conjugate (Jackson Immunoresearch Laboratories, West Grove, PA, USA). The procedure of immunostaining has been described in detail elsewhere [20,57]. The analysis of the slides with spreads was carried out using the fluorescence light microscope Axio Imager D1 (Carl Zeiss, Jena, Germany). A total of 322 spread meiotic nuclei of 60-chromosome mole rats and 108 spread meiotic nuclei of 52-chromosome mole rats were examined.

## 3. Results

### 3.1. Mitotic and Meiotic Chromosomes of 52- and 60- Chromosome Mole Rats

The size, morphology, and G- and C-banding patterns of the sex chromosomes of the mitotic spreads were similar in all Israeli chromosomal forms. Fluorescence in situ hybridization (FISH) of (TTAGGG)_7_ probes revealed telomeric signals of varying intensity at all sex chromosome termini for all chromosomal forms. The X chromosomes showed a weak unstable telomeric signal in the centromeric region. The distribution and intensity of the hybridization signal in the Y chromosome of the 2n = 60 and 2n = 58 forms did not differ from the pattern of telomeric sequences in acrocentric autosomes. The Y chromosomes in the 2n = 54 and 2n = 52 forms presented enlarged blocks of a near-centromeric signal, more prominent than the distal signal in autosomes, and a bright interstitial band of telomeric sequences (Figure 1c,d) [58,59].

**Figure 2:**
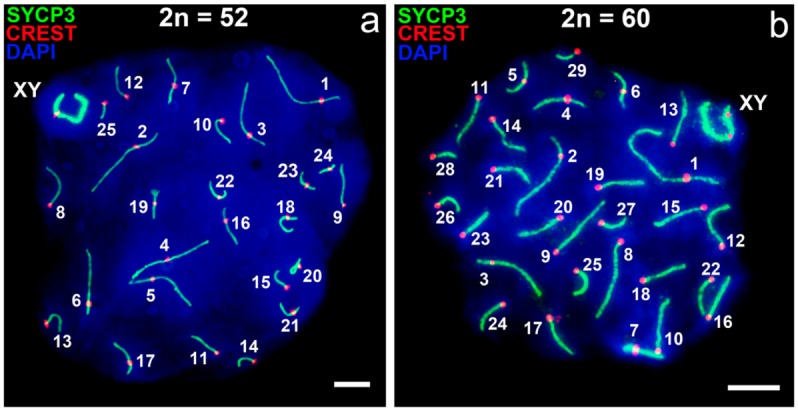
Pachytene spermatocytes of two chromosomal forms of *N. ehrenbergi*. Immunostaining with antibodies to SYCP3 (green) and the centromere (CREST, red). DAPI-stained chromatin (blue). (**a**) Form 2n = 52; 25 synaptonemal complexes (SCs) and XY bivalent are formed; (**b**) form 2n = 60; 29 SCs and XY bivalent are formed. Scale bar (**a**,**b**) = 5 µm.

As predicted, in the spermatocytes of mole rats with 52 chromosomes, 25 autosomal bivalents formed, and thus we were able to distinguish the 25 complete synaptonemal complexes (SCs) and the sex bivalent at the pachytene stage (Figure 2a). The sex chromosomes were moved to the periphery of the meiotic nucleus and formed the so-called sex body (Figure 2a). SCs numbered 1–7 corresponded to large meta- and submetacentric chromosomes, the 8–15 to large and medium acrocentrics, the 16–19 to medium submetacentrics, the 21–24 to small submetacentrics, and the 20 and 25 to acrocentrics. The numbers were identical to the 52-chromosome karyotype [58].

At the pachytene stage, 29 complete SCs and sex bivalents were formed in the spermatocytes of mole rats with 60 chromosomes (Figure 2b). The sex chromosomes were moved to the periphery of the meiotic nucleus and formed the sex body (Figure 2b). SC bivalents denoted 1–7 corresponded to large and medium bi-armed chromosomes and those denoted 8–29 to large, medium, and small acrocentrics. The numbers correspond to the karyotype of 2n = 60 [58].

### 3.2. Synapsis of the Mole Rat Sex Chromosomes

**Figure 3:**
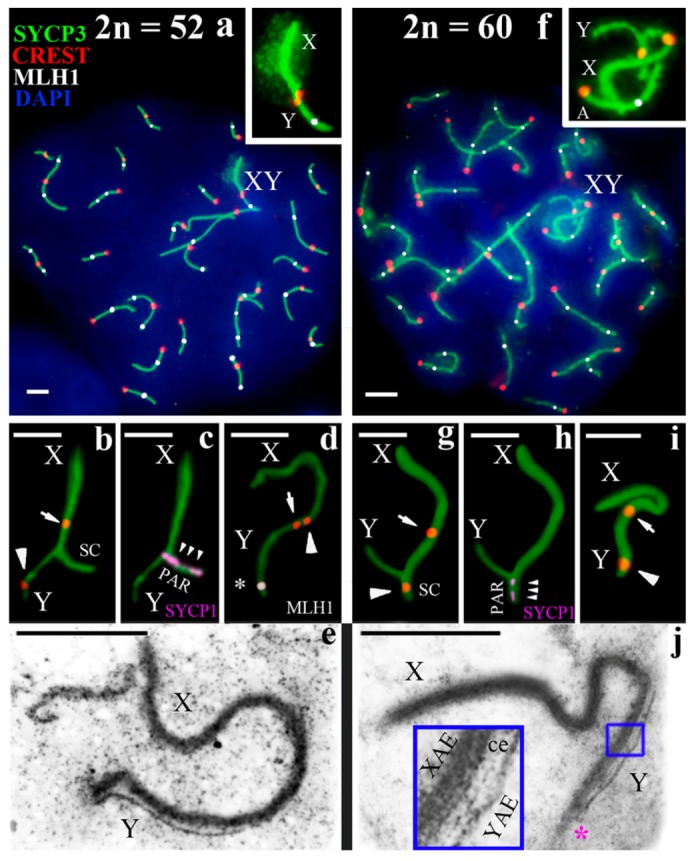
Spermatocytes and sex (X, Y) chromosomes of two chromosomal forms of mole rats *N. ehrenbergi*. (**a**–**e**) Form with 2n = 52, (**f**–**j**) form with 2n = 60. (**а**,**f**) Pachytene spermatocytes of the forms with 2n = 52 (**a**) and 2n = 60 (**f**). The sex chromosomes are enlarged in the insets. A—autosome; (**b**–**e**,**g**–**j**) sex (XY) bivalent at the late zygotene to early pachytene (**b**,**c**,**g**,**h**), and mid pachytene (**d**,**i**) stages; (**a**–**d**,**f**–**i**) The axial SC elements were identified using anti-SYCP3 antibodies (green), the central element with anti-SCP1 antibodies (magenta), the recombination nodules with anti-MLH1 antibodies (white), and anti-CREST antibodies were used for the kinetochores (red). DAPI-stained chromatin (blue). SC: synaptonemal comlex or synaptic cite (**b**,**g**); at sex bivalents, the arrow indicates the centromere of the Х chromosome, and the arrowhead indicates the centromere of the Y chromosome (**b**,**d**,**g**,**i**); the asterisk indicates the MLH1 focus (**d**). The central elements of the SC are formed in the synaptic regions of the sex chromosomes of both mole rats chromosomal forms (the localization of SYCP1 is indicated by three small arrowheads) (**c**,**h**); PAR—pseudoautosomal region; (**e**,**j**) Electron micrographs of a sex bivalent, XY at the mid pachytene stage. The pink asterisk marks synaptic differences in the telomeric sites of X and Y. The blue square marks the site in the inset. XAE denotes axial elements of the X chromosome; YAE denotes axial elements of the Y chromosome; ce denotes the central element. Scale bar (**a**–**j**) = 2 µm.

The analysis of synapsis on the sex chromosomes, following SYCP3 (synaptonemal complex protein 3) and CREST (Calcinosis Raynaud’s phenomenon, Esophageal dysmotility, Sclerodactyly, and Telangiectasia) immunostaining, revealed unexpected results. We found that sex chromosomes of different chromosomal forms, which have almost identical patterns of G- and C-bands, formed sex bivalents of dissimilar configuration. In 60-chromosome mole rats, the synapsis began from the pericentromeric region of the acrocentrical Y, while, in 52-chromosome mole rats, it started at the distal area of the acrocentric Y (Figure 3b–d,g–i). The centromere positions in the Y chromosomes marked these apparently different synapsis (Figure 2 and Figure 3a,f). In the mole rats with 2n = 60, the centromere of the Y in sex bivalents was located at the peritelomeric end of the SC. In the form with 2n = 52, the centromere of the Y laid near the centromere of the submetacentric X chromosome (Figure 3d,i). The SYCP1 antibodies detected the central element of the SC in both forms of mole rats (Figure 3c,h).

Using electron microscopy, we detected no significant differences in the synapsis of the sex chromosomes in the spermatocytes of the 52- and 60-chromosome forms. At the mid pachytene stage, the synapsis between the X and Y extended over the entire Y chromosome in both forms. Additionally, asynaptic and synaptic segments of the X were significantly thicker (3–5 times) than the Y axis (Figure 3e,j, see inset). Previously, the asymmetry in the structure of synaptic segments of the sex chromosomes was termed asymmetric synapsis, and the SC was called asymmetric [60]. It should be noted that there were short-term synaptic differences in the telomere sites of X and Y exclusively in mole rats with 2n = 60 (Figure 3j).

**Figure 4:**
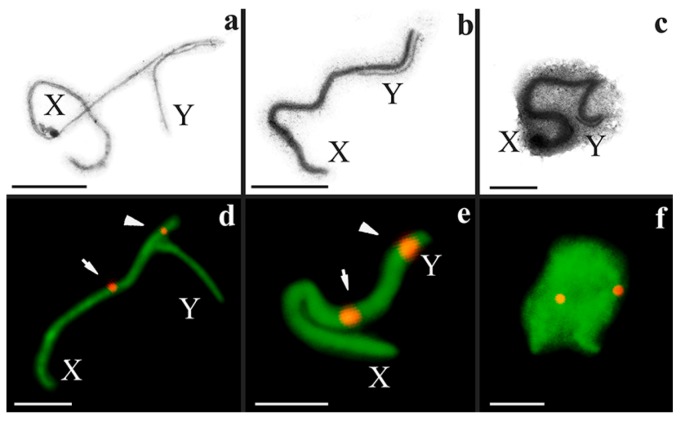
Sex chromosomes of *N. ehrenbergi* with 2n = 60 at different stages of meiotic prophase I. (**a**–**c**) Electron micrographs of a sex bivalent; (**d**–**f**) light microphotos of sex bivalents. Immunostaining with antibodies to SYCP3 (green) and to the centromere (CREST, red); (**a**,**d**) late zygotene to early pachytene stages; (**b**,**e**) mid pachytene stage; (**c**,**f**) late pachytene stage. At sex bivalents, the arrow indicates the centromere of the Х chromosome; the arrowhead indicates the centromere of the Y chromosome. Scale bar (**a**–**f**) = 2 µm.

The synaptic dynamics of sex chromosomes in prophase I in the 60-chromosome mole rats deserve special attention. The axial elements of the sex chromosomes were not thickened and formed a short SC at the mid zygotene stage (Figure 4a,d). Perhaps, this synaptic site is a true PAR. Later, the unpaired segment of the Y chromosome was co-aligned with the X chromosome; a synaptic adjustment might be a possible interpretation. At the mid pachytene stage, the thin Y axis lay along the thickened X axis (Figure 4b,e). At the late pachytene and early diplotene stages, the axial elements of the X and Y chromosomes formed a tangle at the periphery of the meiotic nucleus (Figure 2); the tangle was surrounded by electron-dense material. Thus, the sex chromosomes formed the sex body in a way typical of mammals (Figure 4c,f). The SYCP3 signals were distributed throughout the entire sex body at these stages (Figure 4f).

### 3.3. Recombination of the Mole Rat Sex Chromosomes

A DNA mismatch repair protein MLH1 (mutL homolog 1) was localized in late-recombination nodules [61]. In the mole rats studied, MLH1 foci were identified in 53 spermatocytes of the 52-chromosome form and in 207 cells of the 60-chromosome form; one–two MLH1 foci were identified in each arm of autosomal bivalents, both in the terminal and in the interstitial regions at the pachytene stage. Only one small bivalent did not have an MLH1 signal (Figure 3a,f). This was typical for both forms of mole rats.

**Figure 5:**
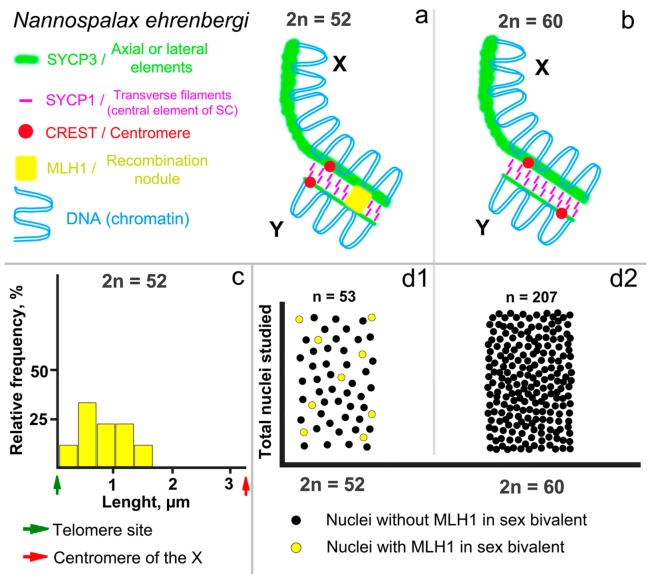
Scheme of synaptic and recombination patterns of mid pachytene XY of *N. ehrenbergi*. Sex bivalent of the 52-chromosome (**a**) and 60-chromosome (**b**) forms of mole rats. The centromeres of the acrocentric Y chromosome of the two forms have different localizations in the sex bivalent. The distribution of MLH1 foci in the synaptic site of the XY bivalent of the 52-chromosome mole rats is shown in the histogram (**c**). The *x*-axis presents the average length of the synaptic site of the sex bivalent in μm; the *y*-axis shows the relative frequency of MLH1 foci in each 10% of the length of the synaptic site of the sex bivalent. The frequency of MLH1 is presented graphically as yellow bars. The data were calculated and presented using GraphPad Prism Version 5.0 (GraphPad Software, San Diego, CA, USA). The total number of nuclei studied in the 52-(**d1**) and 60-(**d2**) chromosome forms of mole rats is represented graphically by black and yellow dots. MLH1 signals in the XY bivalents of the form with 2n = 60 were not detected.

However, the MLH1 foci in the sex bivalents were exposed differently. One MLH1 focus was detected in the sex bivalents in nine of the 53 meiotic nuclei of the 2n = 52 form (Figure 3a,d and Figure 5a,d1). The average distance of the MLH1 signal position was 0.85 ± 0.2 μm (mean values ± standard deviation (SD)) from the telomeric site of the synaptic segment of the sex bivalents in mole rats of the 2n = 52 form (Figure 5c). In the 2n = 60 form, MLH1 foci in the XY bivalent were not detected in any cells immunostained with anti-MLH1 antibodies (Figure 5b,d2), although MLH1 foci were identified in autosomes.

## 4. Discussion

### 4.1. Specific Structure of the Nannospalax ehrenbergi XY Bivalent

Interestingly, mole rat X and Y axes of different thicknesses are involved in synapsis. Such asymmetric synapsis is rare. In most mammals, the synaptic X and Y regions have the same or similar thicknesses [62,63,64,65]. Asymmetric patterns of sex chromosome synapsis have been observed in Norway rat and chinchilla [60,66]. It has been suggested that synapsis occurred between non-homologous chromosomes or non-homologous regions of the sex chromosomes. We believe that the presence of a recombination nodule and a central element in the asymmetric SC of the mole rat sex bivalent indicates at least a partial homology of these segments. Perhaps, these differences in the thicknesses of the pachytene X and Y chromosomes can be explained by the specific accumulation of structural proteins in the SC.

General patterns were manifested in the distribution of recombination nodules in the XY bivalent in the 52-chromosome mole rats. The region with the predominant MLH1 position is located in the peritelomeric site, and there are no such MLH1 foci near the centromere. This pattern has been described in many animals [67,68,69] and in humans [61]. It is worth noting that MLH1 signals in the XY bivalent were identified in 16.9% of the nuclei. The indicator is quite variable in different mammals and can be distinct for several reasons. As we noted earlier [20], the reason for this may be a short period of time when the chromosomes recombine, or a powerful protein cover around the sex chromosomes causing chromatin inactivation that could prevent the penetration of anti-MLH1 antibodies.

Why was an MLH1 focus not identified in the sex bivalent of the 60-chromosome form? We assume that this was due to intercentromere interference. It has long been known that the crossing-over is limited near the centromere [70,71,72]. Along with that, atypical recombination within the near-centromere regions can lead to loss of chromosomes [73]. The suppression of recombination might be due to the presence of pericentromeric heterochromatin [69,74]. As the mole rat X and Y chromosomes have large blocks of C-heterochromatin localized at different ends of the short synaptic segment, this may limit or prohibit the formation of recombination nodules. The evolutionary role of the suppression of recombination, including chromosomal speciation, has been widely discussed [75,76,77,78] and could have occurred in the evolution of mole rats. We cannot exclude that the evolutionary centromere displacement in the Y chromosome could reformat the homologous site of the sex chromosomes. This could lead to a shortening of the segment of true homology, up to the point where the short homologous region can be interstitial, and, as a consequence, to the absence of the MLH1 signal.

### 4.2. Differences in Nannospalax ehrenbergi Sex Chromosomes

The application of the FISH method allowed us to demonstrate different patterns of telomere sequences distribution in the Y chromosomes of Israeli mole rats (Figure 1c,d). Previously, it has been shown that the Y chromosome of the 2n = 52 form differs from the Y of the 2n = 60 form with regard to a bright interstitial CMA_3_ (chromomycin A_3_) band. A thin interstitial hybridization band of species-specific DNA in the Y chromosome of the 2n = 52 form was detected by comparative genomic in situ hybridization [58].

Such differences are confirmed by this study. Here, we have established that the sex chromosomes of the two mole rat forms present different synaptic and recombination patterns. Thus, Israeli *N. ehrenbergi* are marked by two types of the Y chromosome. Our data shed light on the possible ways in which the Y chromosome diverges within this group.

### 4.3. Trends and Possible Ways of Y chromosome Evolution in Nannospalax ehrenbergi

**Figure 6:**
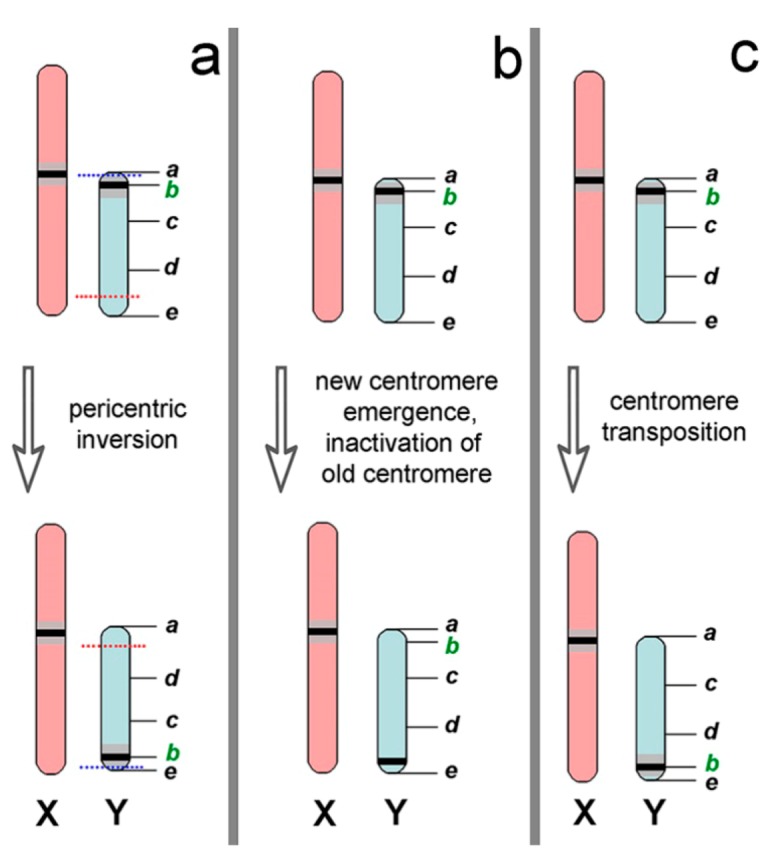
Possible scenarios of the Y chromosome reorganization in the 60-chromosome *N. ehrenbergi*. (**a**) Pericentric inversion changes the position of the centromere in the Y chromosome. The blue and red lines correspond to the points of chromosome breaks; (**b**) formation of de novo centromeres with simultaneous or subsequent inactivation of the old centromere and erosion of C-heterochromatin; (**c**) the centromere, together with heterochromatin, moves to a new position (centromere transposition). The green letter b marks the position of the ancestral centromere.

On the basis of the differences in the location of the centromeres in the male chromosome, we suggest possible scenarios for the reorganization of the Y chromosome. One way is to change the position of the centromere of the Y chromosome through pericentric inversion (Figure 6a). This type of rearrangement takes place in the evolution of the male sex chromosome in some mammals [79]. On the one hand, a large block of C-heterochromatin in the Y chromosome of the 60-chromosome form can indicate this event. Probably, restructuring of the PAR may be supported by synaptic differences in the telomeric regions (Figure 3j). On the other hand, it should cause changes in the G-pattern. However, as was noted above, the Y chromosome of the two chromosomal forms presented almost the same G-pattern [58].

A second variant of chromosome alteration might be as follows. A new centromere in a Y chromosome of the 60-chromosome form could emerge de novo with subsequent inactivation of the old centromere (Figure 6b). As a rule, the new centromere will not form a C-heterochromatin or it will be very small [80], as, for example, in the non-Robertsonian pair of submetacentrics in the *Ellobius tancrei* [21,81]. C-heterochromatin near the old centromere could be eroded. However, the existence of large C-heterochromatin blocks in the Y chromosome conflicts with this scenario.

Perhaps, the most likely scenario is the reorganization of the ancestral Y chromosome through centromeric transposition (Figure 6c). In this case, the centromere moves from one end of the Y chromosome to the other. It is probable that a minimal change in the G-pattern could occur. The centromere could change its position together with C-heterochromatin. We believe that centromeric transposition is a possible mechanism, because the similar data were reported for other species [82,83].

The consequences of the centromeric relocation in the Y chromosome appear to be different in the synaptic and recombination patterns of the sex chromosomes in closely related mole rats. Thus, 60-chromosome mole rats probably demonstrate a unique case of synaptic but achiasmate sex chromosomes. This phenomenon requires further study. To clarify the most probable pathway of the evolution of the Y chromosome, it is important to perform FISH mapping of male sex chromosomes in different mole rat forms, similar to the work of Di Meo et al. [79].

Most likely, the reorganization of the Y chromosome within *N. ehrenbergi* arose after the *Spalax* and *Nannospalax* divergence and independent of autosomal rearrangements (Robertsonian translocations, paracentric inversions, and centromeric shifts). Since the Y chromosomes of all Israeli *N. ehrenbergi*, and even of two of the 60-chromosome forms of *N. xanthodon* from the north of Turkey (*Spalax leucodon sensu lato*), are of the same size and present very similar G- and C-banding patterns, it is probable that the reorganization of the Y chromosome in the Israeli 60-chromosome forms is a relatively recent evolutionary event.

The change in the position of the centromere of the Y chromosome could affect the structure of the PAR. This is confirmed by the fact that we identified a short-term disturbance in the synaptic co-orientation of the X and Y telomeric regions (Figure 3j), as in *Mus musculus*. Two mouse subspecies, *Mus musculus domesticus* and *Mus musculus castaneus*, differ in terms of the shift in the PAR boundary [84]. Restructuring led to a decrease in the recombination level and to the death of meiotic cells in mouse intersubspecific hybrids [85].

It is known that a lack of recombination in autosomes and sex chromosomes can lead to incorrect segregation of chromosomes, formation of unbalanced gametes, and in general a decrease in fertility or even sterility [86]. Aneuploid spermatozoa were previously detected in *M. m. domesticus* × *M. m. castaneus* hybrids, although they retained fertility [85]. It can be assumed that the lack of recombination of the X and Y chromosomes in the 60-chromosome mole rats may have consequences for their successful segregation and increase the number of errors in the formation of mature spermatozoa. However, it is worth noting that recombination, or even synapsis of the sex chromosomes, is lacking in some mammalian species. These species do not have PAR, and their sex chromosomes are always asynaptic, but this does not affect meiotic progression [87]. This phenomenon demonstrates diversity in sex chromosomes evolution.

We hypothesize that only after the autosomal divergence of the mole rat forms, the Y chromosome in the nearest ancestor of the 2n = 52 and 2n = 54 chromosomal forms was subject to insertions or amplification of telomeric sequences [58]. Similar chromosomal reconstructions have been proposed in the evolution of sex chromosomes [88]. The assumption that independent events of duplication or amplification lead to the fixation of different variants of satellite DNA in the autosomes of Israeli mole rat forms [58] confirms our hypothesis. It should be noted that the telomeric pattern has been used as a marker to analyze the inheritance of sex chromosomes in heterozygous mole rats in a hybrid zone [59].

**Figure 7:**
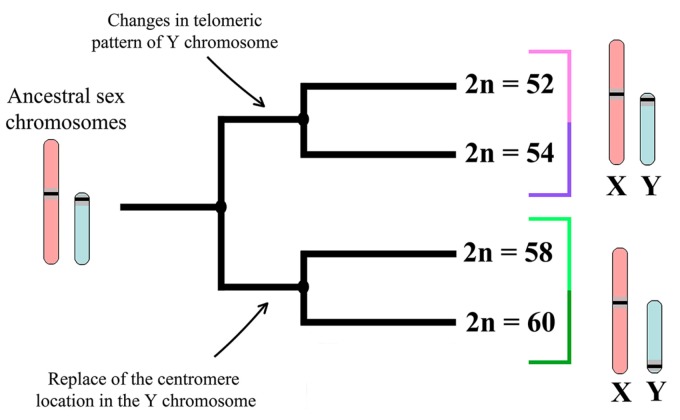
Scheme of sex chromosome differences in Israeli *N. ehrenbergi* based on cytogenetic data.

Existing findings, combined with the data presented here and our unpublished data from the Miilya hybrid zone [89], allow us to conclude that the structural and functional patterns of the Y chromosome in the 52- and 54-chromosome mole rats are identical, just as the Y chromosomes in the 58- and 60-chromosome forms do not differ. We assume that the two groups (“52–54” and “58–60”) were each formed from separate ancestral lines (Figure 7). Possibly, the lines became independent around 280,000 years ago, in accordance with the analysis of allozyme diversity [90] or in the time interval 1.2–2.4 Mya, calculated on the basis of the mean rate of divergence for mammalian mitochondrial DNA [91]. Nevo et al. [92] explained the time differences by the molecular clock or the heterogeneity of the rate of DNA evolution.

What might be the evolutionary fate of the reconstructed Y chromosome in the 60-chromosome form? Perhaps, recombination, the shuffling of genes, reduces the mutation burden. However, the absence of recombination will favor the accumulation of deleterious mutant genes through the Muller ratchet [93], and the Y chromosome will be subject to attrition [23] and degradation [94]. If such a history awaits the male sex chromosome, then undoubtedly it could be a driver of further enhancement of the divergence in mole rats.

A wide autosomal variability might frequently be synchronized or connected with sex chromosome reorganization or even with the emergence of nonclassical systems of sex chromosomes. For example, mice *Mus* possess an incredible variability of autosomes and diverse types of sex chromosomes changes, such as displacement of the PAR border [84], heterochromatin variations [95], Robertsonian translocations [96,97], whole-arm reciprocal translocation (WART) [98], and sex-reverse mutation (XY female) [5]. Considerable chromosomal variations and sex trivalent XY_1_Y_2_ have been described in males with different karyotypes of the common shrew *Sorex araneus* [99,100]. The eastern mole vole has been shown to present unique XX chromosomes in males and females and extreme autosomal variability [4]. Blind mole rats can probably be counted in this group as the reorganization of their Y chromosomes is apparently interconnected with their autosomal changes. Undoubtedly, autosome and sex chromosome variabilities might have a cumulative effect and reinforce the divergence of chromosomal races. Indeed, sex chromosomes rearrangements in heterozygous animals can limit or prohibit the progression of gametogenesis, form aneuploid gametes, and lead to a decrease in fertility [86,101,102,103].

## 5. Conclusions

The results presented here underline the diversity between different karyotypes found in *N. erhenbergi.* We assume that it is most appropriate to divide all Israeli mole rats into two large clusters: ‘2n = 52–2n = 54’ and ‘2n = 58–2n = 60’ (Figure 7). The division into two clusters is supported by the analysis of mitochondrial DNA [91,92,104,105]. Most likely, each cluster corresponds to the species status, and the representatives within each cluster correspond to the level of the chromosomal form. The species status is given for each chromosomal form of Israeli mole rats on the basis of extensive studies (summarized in [48]). However, according to the biological species concept, reproductive isolation is a key issue. Its detailed analysis permits the most adequate discussion of diversification within the different taxa. Wahrman et al. [42] started this field of study, but it should be continued. Studies of meiosis in different forms and their hybrids will help to decipher the mechanisms of reproductive isolation. To identify signs of meiotic arrest and sterility, it is necessary to study the features of meiotic progression and gametogenesis in ‘inter-cluster’ and ‘intra-cluster’ mole rat hybrids.

To date, meiotic studies of the structure and behavior of the sex chromosomes have confirmed the deep diversification in the *N. ehrenbergi* superspecies complex and the prospect for evolutionary studies in the mammalian group.
